# Risk Behaviors, Prevalence of HIV and Hepatitis C Virus Infection and Population Size of Current Injection Drug Users in a China-Myanmar Border City: Results from a Respondent-Driven Sampling Survey in 2012

**DOI:** 10.1371/journal.pone.0106899

**Published:** 2014-09-09

**Authors:** Lei Li, Sawitri Assanangkornchai, Lin Duo, Edward McNeil, Jianhua Li

**Affiliations:** 1 Yunnan Institute of Drug Abuse, Kunming, Yunnan, P.R. China; 2 Epidemiology Unit, Faculty of Medicine, Prince of Songkla University, Hat Yai, Songkla, Thailand; 3 HIV/AIDS Asia Regional Program Yunnan Management Office, Kunming, Yunnan, P.R. China; Johns Hopkins School of Public Health, United States of America

## Abstract

**Background:**

Injection drug use has been the major cause of HIV/AIDS in China in the past two decades. We measured the prevalences of HIV and hepatitis C virus (HCV) prevalence and their associated risk factors among current injection drug users (IDUs) in Ruili city, a border region connecting China with Myanmar that has been undergoing serious drug use and HIV spread problems. An estimate of the number of current IDUs is also presented.

**Methods:**

In 2012, Chinese IDUs who had injected within the past six months and aged ≥18 years were recruited using a respondent-driven sampling (RDS) technique. Participants underwent interviews and serological testing for HIV, HBV, HCV and syphilis. Logistic regression indentified factors associated with HIV and HCV infections. Multiplier method was used to obtain an estimate of the size of the current IDU population via combining available service data and findings from our survey.

**Results:**

Among 370 IDUs recruited, the prevalence of HIV and HCV was 18.3% and 41.5%, respectively. 27.1% of participants had shared a needle/syringe in their lifetime. Consistent condom use rates were low among both regular (6.8%) and non-regular (30.4%) partners. Factors independently associated with being HIV positive included HCV infection, having a longer history of injection drug use and experience of needle/syringe sharing. Participants with HCV infection were more likely to be HIV positive, have injected more types of drugs, have shared other injection equipments and have unprotected sex with regular sex partners. The estimated number of current IDUs in Ruili city was 2,714 (95% CI: 1,617–5,846).

**Conclusions:**

IDUs may continue to be a critical subpopulation for transmission of HIV and other infections in this region because of the increasing population and persistent high risk of injection and sexual behaviours. Developing innovative strategies that can improve accessibility of current harm reduction services and incorporate more comprehensive contents is urgently needed.

## Introduction

Injection drug use, particularly heroin, has been the major cause of HIV/AIDS in China over the past two decades [1]. Although in recent years heterosexual contacts have become the dominant mode of transmission among newly diagnosed HIV cases, the majority of the cumulative HIV/AIDS infections is still attributed to the epidemic of risky injection behaviors [2]–[4]. At the end of 2009, an estimate of 740,000 people was living with HIV/AIDS in China. Of the 48,000 new HIV infections, 24.3% were attributed to injection drug use. The reported national HIV prevalence among injection drug users (IDUs) was 9.3%, with higher rates at provincial levels, including 29.0% in Xinjiang, 25.0% in Guangxi and 18.3% in Yunnan, respectively [5].

Ruili is the biggest national-level trading port with Myanmar. It is situated in Dehong prefecture in the western part of Yunnan province and shares a border with Myanmar on the southwest, southeast and northwest of 169.8 km. Over the past 25 years, Ruili's geographic location and expansive economic activities ranging from jade to drugs has caused severe public health problems, especially the transmission of HIV/AIDS [6]. Since the first HIV outbreak in China was identified among IDUs in Ruili in 1989, this area has become one of the most severely affected locales in the whole country [7], [8] with a reported cumulative total of 4554 HIV/AIDS cases up to 2009, and 41.8% transmitted by injection drug use [9].

Monitoring HIV and other blood-borne or sexually transmitted infections (STI) and related risky behaviors among IDUs is very important because they are considered as a key bridge population for the spread of these diseases from high-risk groups to the general population [10]. Because of inherent social stigmatization and vulnerability of IDUs that may cause many difficulties to find or contact people among this population, although there have been previous studies about IDUs conducted in Ruili city, nearly all of them included participants recruited by convenience sampling from institutions, such as detoxification/detention centers or methadone maintenance treatment (MMT) clinics, or used secondary data from different sources such as sentinel surveillance sites [8], [11], which are unlikely to provide representative samples of the targeted populations. In addition, to our knowledge, a reliable estimate of the number of IDUs is still lacking in this border region. To fill these gaps, we conducted a biological and behavioral survey using respondent-driven sampling (RDS), which is a chain-referral sampling method for hard-to-reach populations and, theoretically, can generate a sample that is much more representative than other sampling techniques [12]. The study aimed to identify the prevalence and the correlates of HIV and hepatitis C (HCV) amongst Chinese IDUs in this border city. We also used the multiplier method, which is an indirect approach for estimating population size of hidden populations by combining available service data and findings from our survey to estimate the number of current IDUs in Ruili city [13]. We present our findings here, and hope to provide conclusive evidence for health officers and policy makers for planning programmatic and policy responses, guiding allocation of resources for prevention and intervention, as well as monitoring and evaluating current implementation of these strategies in a border city which has undergone an explosive HIV spread and high HIV prevalence.

## Methods

### Participants and recruitment

A cross-sectional survey (including interviews and serological testing) was conducted among IDUs aged 18 years or older in Ruili city, Yunnan, China between May and June 2012. An IDU was defined as an individual who had injected any illicit drug within the previous six months before the interview. To obtain a representative sample of Ruili people, participants were also required to be Chinese and had resided in or worked in Ruili for longer than six months at the time of their interview.

Based on recommendations obtained from a prior meeting with local public health staff and outreach workers, our RDS recruitment center was set in a drop-in center of a needle-syringe exchange program (NSP) run by HIV/AIDS Asia Regional Program (HAARP) Yunnan Management Office and local government. The NSP staff have good rapport with local IDUs and are well trusted by them. One female and two male IDUs of three different age-groups (≤25, 26–35 and >35 years) and three different ethnic groups (Han, Jingpo and Dai) were selected as ‘seeds’ of the RDS chain. Each seed was given three uniquely coded coupons, which was valid for 30 days, to recruit his/her peers. Individuals who could present a valid coupon before the due date and who were eligible for this study were enrolled, and successively distributed, but only up to three recruitment coupons to recruit their peers. The recruitment process continued until the required sample size (at least 362) and equilibrium with respect to the main variables being measured was achieved. Using a RDS method, once the ‘equilibrium’ is reached, the sample compositions will be stable and be independent of the initial participants (seeds).

Participants were compensated with 50 Chinese Yuan (about 8 US dollars) for their participating in the study and an additional 20 Chinese Yuan (about 3 US dollars) for successful recruitment of each eligible participant from their peer network. All interviews were conducted by the principle researcher of this study (LL) and two well-trained indigenous outreach workers of the NSP drop-in center. The participants were initially screened for their injection drug use by checking track-marks and asking some questions about drug use. A blood sample was then drawn for serological testing.

### Measures

Items in the questionnaire included demographic information, substance use history, injection behaviors, sexual behaviors, utilization of harm reduction services and lifetime history of incarceration. In addition, information about social network size that was required for RDS purpose in order to reduce the bias that may occur through oversampling of homogenous networks was also collected [14].

Participants were asked about their lifetime and current (in the past 6 months) history of drug use and injection behaviors. Drug use history included the types of substances used (including alcohol and prescription drugs used illegally) and initial age of use. Injection behaviors investigated in this study included age at initiation, types of substances injected, frequency of injection, history of sharing and use of used needles/syringes and other injection equipment.

For sexual behavior, participants were asked how many sex partners they ever had both in their lifetime and the past 6 months. They were also asked if they had regular (spouses, boy/girl friends) or non-regular partners (casual sex partners, sex workers/sex clients) in the past 6 months, and the frequency of condom use with different types of partners. Other variables included age at sexual debut, engaging in sex under the influence of any drugs (past 6 months), and history of exchanging sex for drugs.

Lifetime and current utilization of harm reduction services were measured by 11 yes-no items regarding education and counseling/testing about HIV/AIDS and drug use, safe injection and proper condom use skills, and access to free condom distribution, MMT and needle-syringe exchange programs.

### Laboratory Testing

Each participant provided 7 ml of intravenous blood for serological testing of HIV, hepatitis C virus (HCV), syphilis antibody and hepatitis B virus (HBV) surface antigen (HBsAg). All samples were sent to a laboratory of the Center for Disease Control and Prevention (CDC) in Ruili city for initial testing.

HIV screening was conducted using two enzyme-linked immunoassays (ELISAs; Livzon Group Reagent Factory, Zhuhai, China). If both tests were reactive, a western blot test (WB test; Genelabs Diagnostics Pte Ltd., Singapore) was conducted for confirmation at the HIV Confirmatory Laboratory in CDC of Dehong Dai and Jingpo Autonomous Prefecture. The sample was considered HIV positive when both the ELISA and WB tests were positive. The presence of HBsAg and anti-HCV antibody was detected by ELISA (ELISA; Beijing Wantai Biologic Production Co. Ltd, Beijing, China). Syphilis screening was performed by rapid plasma regain (RPR; Beijing Wantai Biologic Production Co. Ltd, Beijing, China). Specimens testing positive for syphilis antibody were confirmed by the treponema pallidum particle agglutination test (TPPA; Livzon Group Reagent Factory, Zhuhai, China), with titer ≥1∶8 being considered as current infection and otherwise past infection.

Serological specimens and testing results were anonymous and were only linked to the survey data by a unique coupon number. All participants were asked to return or make a phone call after three weeks to obtain their results by providing both the unique coupon number and their date of birth. Pretest counseling was provided to all participants, while posttest counseling and referrals were provided to those who were serologically positive.

### Multiplier method for estimation of IDU population size

As one of the indirect approaches for estimating the size of hidden populations, the multiplier method has been increasingly used in different settings in recent years because of its simplicity in implementation [15], [16]. Use of the multiplier method depends on the availability and quality of data collected from two sources. The first source, known as “benchmark data”, is usually obtained from institutions or intervention programs while the second source, namely “multiplier data”, is usually obtained from a behavioral survey of a target population based on a probability sampling technique [13]. These two data sources are also required to be independent of each other, and somewhat overlap in a known way, that is, the population being counted has a probability to be included in either or both sources [17]. Once meeting these assumptions, the population size can be calculated from the formula: 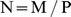
where N is the size of population being estimated, M is the benchmark data, which can be the number of subjects in a target population who have accessed services in selected institutions or the intervention programs over a specified time frame, and P is the proportion of the target population who report a utilization of corresponding services during the same period.

To fulfill the major assumptions mentioned above, we obtained our benchmark data from a local MMT clinic. In China, the eligibility criteria of MMT include age at least 20 years, being a permanent local resident or having resided in this place more than six months, having had a history of multiple unsuccessful treatment, having received compulsory detoxification treatment at least twice or rehabilitation through labor treatment at least once [18]. To avoid duplication of data, only the newly admitted IDUs (including new admissions and readmissions) in the past six months prior to the RDS survey (before May 2012) in the MMT clinic in Ruili city were counted. To estimate the proportion of IDUs who reported an admission to the MMT clinic during the same period, the respondents in our RDS survey were asked if they were enrolled in the local MMT clinic during November 2011 to April 2012.

### Statistical analysis

For adjusting potential bias arising from RDS due to respondents' different personal social network size and homophily of recruitment, RDSAT software (Respondent-Driven Sampling Analysis Tool; version 5.6.0; www.respondentdrivensampling.org), which is specifically designed to analyze data collected through RDS, was used for descriptive analyses and to calculate sampling weights for all univariate and multivariate analyses. The weighted prevalence of HIV, HBV, HCV and syphilis, drug use and injection behaviors, sexual behaviors, and coverage of harm reduction services in the different time periods were described using mean and standard deviation (SD), median and interquartile range (IQR), or frequency and percent as appropriate. Adjusted point estimates and 95% confidence intervals (CI) were given for all variables.

Univariate and multivariate logistic regression models were performed to identify factors associated with HIV and HCV infections, using survey design-based methods in the R language and environment (version 2.13.0) [19]. Independent variables associated with the outcome at p-value<0.10 in univariate analysis were considered for inclusion in the initial multivariable model and further refinement of the model was done by a backward elimination procedure. At each step, the variable with the largest p-value from the Rao-Scott likelihood ratio test was removed until all variables remaining in the final model had a p-value<0.05.

### Ethics statement

Written informed consent was obtained from all participants before being interviewed. All information was kept strictly confidential and was used for research purposes only. All data were recorded and analyzed anonymously. This study was approved by the Human Studies Committee of Yunnan Institute of Drug Abuse (YIDA), China, and the Ethics Committee of the Faculty of Medicine, Prince of Songkla University, Thailand.

## Results

A total of 370 eligible participants were recruited after 11 waves. Equilibrium was achieved by wave 7 with regard to some key variables such as gender, age, ethnicity, marital status, education level, occupation, residential status, and infection rates of HIV, HBV, HCV and syphilis.

### Social-demographics, utilization of services and prevalence of HIV/HBV/HCV/syphilis

Almost all participants were male with a mean age of 36.2 years (SD = 9.4). Most were local residents of Ruili with more than half being from an ethnic minority such as Jingpo and Dai. Over one-third were single and most had below junior high school level of education. Most participants had a current job with one-third each working as a farmer or casual laborer. Almost 80% reported having a history of incarceration in their lifetime. (Table 1)

**Table 1:** Social-demographic characteristics of injection drug users (IDU).

	Weighted %	95% CI^a^
**Age**		
≤25 years	13.6	(9.6–17.9)
26∼35 years	36.7	(30.9–42.4)
>35 years	49.7	(43.7–56.1)
**Male**	92.7	(89.3–95.8)
**Ethnicity**		
Han	45.7	(37.2–53.2)
Others	54.3	(46.8–62.9)
**Marital status**		
Single	38.4	(32.6–43.8)
Living with a spouse or partner	35.5	(30.2–41.4)
Divorced/separated/widowed	26.1	(21.7–30.9)
**Education**		
Primary school and below	49.3	(43.2–55.5)
Junior high school	38.9	(32.9–44.9)
Senior high school and above	11.8	(8.7–15.5)
**Occupation**		
Casual laborer	34.3	(29.2–41.5)
Farmer	38.3	(30.4–45.6)
Others	11.7	(8.5–14.8)
Unemployed	15.6	(11.6–19.0)
**Monthly income ≤1500 RMB**	65.5	(60.7–70.7)
**Residential status**		
Local resident	84.9	(80.9–88.4)
Non-local	15.1	(11.6–19.1)
**Having a history of incarceration**	78.3	(73.3–83.3)

a 95% CI = 95% confidence interval.

Half (49.3%) of the participants reported having received more than six types of prevention services during their lifetime with 53.4% reporting using any of these services in the past 6 months. Lifetime utilization of NSP and MMT was reported by 32.0% and 11.4% of participants, respectively while 28% and 3.5% reported having used NSP and MMT services in the past six months. The estimated prevalence of HIV, HBV, HCV, and current syphilis-infection was 18.3%, 9.8%, 41.5%, and 7.5% respectively.

### Drug use and injection behaviors

The mean age at first use of any substances (excluding alcohol) was 23.3 (SD = 3.7) years, with up to 26.4% starting before the age of 18 years. Heroin, alcohol and amphetamine-type stimulants (ATS) were the three most prevalent substances used by participants in their lifetime. Use of other substances included opium, benzodiazepines, marijuana, ketamine, Demerol and cocaine. About 70% of the participants had used at least three kinds of substances in their lifetime with a range of 2–8 kinds (median  = 4, IQR = 3, 5). In the past 6 months, besides heroin, the most frequently used drug was ATS, followed by alcohol, benzodiazepines and opium. (Table 2).

**Table 2:** Lifetime and current (past six months) use of different drugs among IDUs.

	Lifetime use		Current use	
Drug type	Weighted %	95% CI^a^	Weighted %	95% CI^a^
Heroin	100	-	100	-
Alcohol	95.5	(93.6–97.3)	60.1	(54.0–66.1)
Amphetamine-type stimulants (ATS)	84.2	(79.9–89.3)	75.2	(70.0–81.1)
Opium	60.7	(54.8–66.9)	9.3	(5.7–13.3)
Tranquilizers/barbiturates/benzodiazepines	35.4	(30.4–40.6)	22.1	(18.5–26.2)
Other drugs (marijuana, ketamine, Demerol, cocaine)	12.2	(8.5–16.3)	3.2	(1.3–5.7)

a 95% CI = 95% confidence interval.

The mean age at first drug injection was 28.9 years (SD = 8.8) with 8.4% starting before the age of 18 years. Almost half (45.8%) had injected drugs for more than five years (mean 7.2 years, SD 6.4). Heroin, diazepam and Demerol were the most common drugs injected by participants in their lifetime (100%, 34.2% and 3.9%, respectively). Of all, 36.1% had injected more than one type of drug and 27.1% and 62.3% had shared needle/syringe and other injection equipment such as bottles, spoons, cotton, filters, solution or rinsing water from a shared container with others in their lifetime.

In the past six months, 77% of participants reported injecting drugs daily while the rest did so monthly or weekly. The most common drugs of use were heroin (100%), diazepam (21.8%) and Demerol (2.1%). Receptive needle/syringe sharing accounted for 9.6%, distributive sharing 12.3% and other equipment sharing 34.1% of all participants. Sixty-two percent reported ever injecting with a previously self-used needle or syringe.

### Sexual risk behaviors

Nearly all participants (367) reported having a sexual encounter in their lifetime. Amongst those who had ever had sex, the mean age at sexual debut was 18.9 years (SD = 3.2), and 53.1% were sexually active before 18 years of age. The majority (83.5%) reported having two or more sex partners in their lifetime.

In the previous six months, 51.7% of the participants reported having sexual intercourse, including 17.6% who had more than one partner. Of these, 82% had a regular partner in the past six months and 93.2% of them reported having unprotected sex with this type of partner. Having sex with non-regular partners was reported by 33.6% of participants and among these 69.6% reported inconsistent condom use with these partners. Twenty-eight percent of those who ever had sex used a condom during their last sexual encounter.

Having sexual behaviors when high on drugs was reported by 57.0% of participants who had sex in the past six months. Exchanging sex for drugs was reported by 2.4% of participants.

### Factors associated with HIV status

In the univariate analysis, variables marginally associated with the outcome variable of HIV sero-status (p<0.10) included age-group, HCV infection, syphilis infection, history of incarceration, duration of drug injection, number of drugs injected, needle/syringe sharing and reusing in lifetime and past six months, frequency of injection and condom use with non-regular partners. In the multivariate analysis, HCV infection, having a history of injection drug use for more than five years and having experience of sharing needles and syringes with others during their lifetime were independently and significantly associated with being HIV positive. (Table 3, Table 4).

**Table 3:** Factors associated with HIV and HCV infections in univariate analysis.

	HIV positive				HCV positive			
	Weighted %^a^	OR^b^	(95% CI)^c^		Weighted%^a^	OR^b^	(95% CI)^c^	
**Age**								
≤25 years	6.4	1			30.3	1		
26∼35 years	23.4	1.19	(1.05–1.35)	f	48.0	1.19	(0.97–1.45)	
>35 years	17.8	1.13	(1.02–1.25)	f	40.0	1.12	(0.93–1.35)	
**Having a history of incarceration**								
No	6.0	1			30.8	1		
Yes	21.8	1.17	(1.08–1.27)	f	44.5	1.16	(0.99–1.34)	e
**HIV positive**								
Negative	_				32.3	1		
Positive					83.6	1.67	(1.42–1.95)	f
**HCV positive**								
Negative	5.2	1			_			
Positive	37.1	1.37	(1.23–1.52)	f				
**Syphilis positive**								
Negative	17.4	1			41.0	1		
Positive	34.3	1.21	(0.99–1.48)	e	54.7	1.13	(0.92–1.40)	
**Duration of injection drug use**								
≤5 years	5.7	1			33.8	1		
>5 years	32.1	1.14	(1.04–1.24)	f	51.0	1.2	(1.05–1.36)	f
**Number of drugs injected**								
1	12.3	1			34.6	1		
≥2	28.2	1.17	(1.05–1.30)	f	53.2	1.21	(1.06–1.37)	f
**Sharing needle/syringe**								
No	7.4	1			36.5	1		
Yes	47.5	1.5	(1.31–1.71)	f	53.5	1.22	(1.05–1.42)	f
**Sharing other injection equipment**								
No	14.8	1			30.6	1		
Yes	21.0	1.07	(0.97–1.18)		47.7	1.19	(1.05–1.35)	f
**Frequency of injection drug use** d								
Monthly/weekly	8.8	1			29.4	1		
Daily	20.3	1.12	(1.01–1.23)	f	43.9	1.17	(1.01–1.36)	f
**Receptive needle/syringe sharing** d								
No	15.3	1			38.3	1		
Yes	47.5	1.39	(1.17–1.66)	f	74.2	1.43	(1.22–1.66)	f
**Injection with a previously self-used needle/syringe** d								
No	8.6	1			32.2	1		
Yes	24.4	1.17	(1.07–1.28)	f	46.8	1.18	(1.03–1.35)	f
**Having unprotected sex with regular sex partner** d								
No	19.4	1			10.0	1		
Yes	12.1	0.93	(0.74–1.18)		40.8	1.42	(1.24–1.63)	f
**Having unprotected sex with non-regular sex partner** d								
No	8.2	1			33.2	1		
Yes	18.9	1.31	(1.05–1.64)	f	52.1	1.17	(0.85–1.62)	

a Row percents.

b OR = Odds ratio.

c 95% CI = 95% confidence interval.

d Past 6 months.

e p<0.1.

f p<0.05.

**Table 4:** Final model showing association of factors with HIV sero-prevalence among IDUs.

	AOR^a^	(95% CI)^b^	*P* value
**HCV positive**	1.29	(1.17–1.43)	<0.001
**Duration of injection drug use >5 years (ref.≤5)**	1.25	(1.13–1.39)	<0.001
**Shared needle/syringe (lifetime)**	1.45	(1.27–1.65)	<0.001

a AOR = Adjusted odds ratio.

b 95% CI = 95% confidence interval.

### Factors associated with HCV infection

Variables associated with HCV infection in the univariate analysis included HIV status, history of incarceration, duration of injection drug use, numbers of drugs used, needle/syringe and other equipment sharing in lifetime and past six months, reusing needle/syringe, frequency of injection and condom use with regular partners. Four variables were found to be independently associated with HCV infection in the final model, namely HIV infection, having injected more than one type of drug during lifetime, having shared other injection equipment in the past and having unprotected sex with regular sex partners within the past six months. (Table 3, Table 5).

**Table 5:** Final model showing association of factors with HCV sero-prevalence among IDUs.

	AOR^a^	(95% CI)^b^	*P* value
**HIV positive**	1.59	(1.36–1.87)	<0.001
**Number of drug types injected ≥2 (lifetime)(ref.1)**	1.14	(1.01–1.29)	<0.05
**Shared other injection equipment (lifetime)**	1.15	(1.03–1.30)	<0.05
**Had unprotected sex with regular sex partner (past six months)**	1.44	(1.23–1.68)	<0.001

a AOR = Adjusted odds ratio.

b 95% CI = 95% confidence interval.

### Estimated IDU population size

Our RDS survey revealed that the proportion of those who reported having an admission in the MMT clinic between November 2011 and April 2012 was 2.8% (95% CI: 1.3–4.7%).The number of IDUs who were newly admitted or re-admitted in the MMT clinic during the same time period was 76, thus the total number of IDUs based on this data source was estimated to be 2714 (95% CI: 1617–5846). Based on the census data in 2011, the total population of Ruili City was 181,239 (including unregistered citizens), the prevalence of IDUs was thus estimated to be 2714/181,239 = 1.50% (range 0.89%–3.23%).

## Discussion

Overall, our findings indicate a decrease in HIV prevalence (18.3%) among current IDUs compared with 57.6% reported by a study conducted in the same area in 2005 using several data sources [8]. The decrease is also supported by an investigation about trends in the HIV epidemic in Yunnan province which found that the provincial average prevalence rate among IDUs decreased from 32.4% in 2004 to 28.4% in 2007 after a long-term increase since 1992 [4].

Similarly, the rates of lifetime (27.4%) and recent needle/syringe sharing (9.6% for receptive sharing and 12.3% for distributive sharing) among current IDUs were found to be lower than prior studies. For example, a cross-sectional survey conducted among 2080 IDUs recruited from various communities, VCT clinics, NSP and MMT programs in five different regions of Yunnan province in 2009 reported an average level of needle/syringe sharing of 33.7% [20]. Some of these differences may be attributed to different sampling techniques or data sources used. Previous studies mostly utilized convenience sampling based on venues and institutions such as clinics, detention places or detoxification centers causing an oversampling of IDUs with more serious problems. Moreover, harm reduction services have started to show its positive effects on preventing HIV infection by reducing needle and syringe sharing in this group. However, it is also possible that needle/syringe sharing behavior is under-reported because of stigmatization [21].

Despite the above positive findings, injection risk behaviors in these IDUs remain a serious concern. The identified relationship between HIV infection and duration of injecting drugs is supported by previous studies and suggests the importance of developing strategies to delay the initiation of injection drug use [22]. Among our participants who were HIV sero-positive, more than two-thirds (55/70) had ever shared needles/syringes in their lifetime, and nearly one-fifth (13/70) had distributed needles/syringes to other IDUs in the past six months, posing a potential risk for HIV transmission to their syringe-sharing peers. In addition, the association between HIV and lifetime needle/syringe sharing among the current IDU population, and the fact that more than half of participants reported reusing their own syringes during the past six months, reflects an inadequate access to clean needles/syringes at present. The poor coverage of NSP for IDUs (only 32% lifetime coverage and 28% in the past six months), compared to the expected goal of 50% in 2010 by the central government [23], underpins these results. Barriers of access to NSP among IDUs could possibly be distance from home to the NSP site, limited operating times, strict regulations such as 1-for-1 exchange and fear of being arrested. All of these barriers could result in a low coverage of service and make other venues, such as pharmacies, a more attractive source of needles/syringes [24]. Like other cities in China [25], our conversations with local IDUs in Ruili revealed that pharmacies were used as an option to obtain sterile injecting equipment as its sale is legal (personal communication with local injection drug users, August, 2012). However, living in mountainous areas and costs may restrict this source. Based on this local situation, we emphasize the need for increasing the accessibility of sterile injection equipment, such as strengthening current mobile NSP services through outreach workers and trialing a pharmacy-based needle/syringe exchange program [26].

Another point of concern found in this study is the low coverage of MMT among IDUs. Previous studies suggest that at least 60% coverage is required for effective interventions to reverse or stabilize the HIV epidemic among drug users [27]. Only 11.4% of our participants had ever engaged in MMT, which seems far too low to have a sufficient impact on the HIV epidemic. Similar to the challenges faced by NSP, the geographical terrain and the cost of transportation pose difficulties to IDUs from remote areas to utilize MMT services. Although current established mobile services have helped with this issue to a certain extent [28], for higher coverage, the program might need to explore more flexible and effective strategies. Besides, eligibility criteria required by Chinese MMT undoubtedly may deter IDUs who actually could have benefited from it, including unexposed or migrants. Approximately one sixth of our participants were non-local residents and none of them reported ever being enrolled in a local MMT clinic. Migrant status has been proven to facilitate vulnerability to drug use and transmission of infectious diseases due to the relevant economic and survival issues, social isolation, lack of knowledge or proper documentation to access local services, and fear of deportation or arrest [29]. Nowadays, the lack of migrant-targeted MMT policies remains a major concern in China. Greater efforts must be made to achieve the targeted outcome of harm reduction.

Apart from risky injection behaviors, sexual risk behaviors of these IDUs, including having multiple sex partners and unprotected sex, remained high and were associated with HIV infection. Among these IDUs, the prevalence of consistent condom use was very low, especially with regular sex partners, which is consistent with previous studies among drug users in China [30]. The reasons for inconsistent condom use reported in previous studies included feeling uncomfortable during sexual intercourse, believing that their partners were not infected with HIV and other STIs, or perceiving condom use as a mistrustful behavior within an established relationship [30]. These reasons may also be applied to our study and may be due to a lack of effective behavioral intervention, especially designed for changes of sexual risk behaviors related to HIV infection in the current policies [31]. Accordingly, theory-based intervention programs have proven to be effective in other countries, including psychological and behavioral counseling services, and these should be integrated within existing harm reduction programs to promote behavior change among IDUs in China [31].

As a major cause of chronic liver disease worldwide and a potential contributor to morbidity and mortality [32], the epidemic of viral hepatitis could not be overlooked in this study. The HCV infection rate among current IDUs in our study was moderate (41.5%), compared to other studies conducted in China [33] and in South and Southeast Asia (range 10–100%) [34]. Our finding of a positive association between HIV and HCV infection is consistent with two previous studies among IDUs [33], [35]. The explanation may be that the two infections have shared routes of transmission and that HIV infection can make the host more vulnerable to HCV infection by increasing both viral infectiousness and viral susceptibility [36]. Our finding also supports a previous study which found an association between sharing injection paraphernalia, such as cookers and filtration cotton, and HCV sero-positive [37]. In the past several decades, the prevention strategies for HIV infection among IDUs emphasized only on reducing needle/syringe sharing. However, this appears to be inadequate and less effective against HCV [38]; low awareness of transmission risks associated with indirect share of injection paraphernalia still remains. This calls for specific preventive strategies and education including distribution of injection preparation equipment in the NSP and providing HCV counseling and testing services. Moreover, given a significant association between HCV and unprotected sex with regular sex partners, interventions to reduce the risk of HCV transmission from IDUs to their sexual partners are needed, as suggested by Terrault and Shang et al [39], [40]. The prevalence of HBV infection among our participants is consistent with previous results from global systematic reviews which revealed that the global prevalence of HBsAg among IDUs was 8.4%, ranging from 3.5% to 20% [41]. Similar to the case of co-infection between HIV and viral hepatitis [42], co-infection with HBV and HCV is likely to further increase the risk of liver disease complications and attendant mortality [43], [44]. Given the comparable prevalence of HIV, HCV and HBV infections in this IDU population, provision of vaccination against HBV for all susceptible IDUs, especially for those who are already infected with HIV and HCV, should be taken into consideration in future prevention and intervention efforts.

Another interesting finding in this study was a particularly high level of lifetime ATS use (84.2%) among IDUs compared to other studies [45]. This may be attributed to the special geographic location of this border region, which is an important transit point for major methamphetamine trafficking from Myanmar to China and abroad. As reported in other studies [46], ATS use by IDUs poses more harm, especially from high-risk sexual behaviors. This is a serious concern and needs further research to describe the contexts, associated behaviors, and health outcomes of ATS use among these primary heroin IDUs.

Our estimate of 2714 (95% CI: 1617–5846) IDUs in Ruili is much higher than the 1650 (95% CI:1500∼1800) estimated for the same area in 2005 [8]. Based on our representative sample, and an increasing population of drug addicts as well as increased proportion of IDUs at the national level [5], our higher estimate is likely to be more convincing. Comparing our estimated prevalence of IDUs (1.50%, range: 0.89–3.23%) to a population-weighted estimate of 0·36% from a systematic review (range of 0·056% in South Asia to 1·50% in Eastern Europe) [47], our figure signals a major health concern for Ruili city.

Our study has several limitations. First, self-reported data, especially on sensitive questions such as drug use and sexual behaviors, may have resulted in an under-reporting of the real situation. Second, the lifetime and six-month recall period might have caused recall problems for some participants. Third, we do not know how many IDUs (if any) refused to join this study. Although the RDS method can theoretically provide a representative sample of a target population once the equilibrium is achieved, we do not know if there were any differences between our respondents and the possible non-respondents.

Despite these limitations, this is one of the few studies conducted among IDUs in China that used a representative sample and applied appropriate statistical methods to correct for differential recruitment bias. It provides valuable insights into the current IDU population and will be useful for public health planning and evaluation efforts. Our findings suggest that IDUs may continue to be a critical subpopulation for transmission of HIV and other infections in this region because of the increasing population and persistent high risk of injecting and sexual behaviors [48]. However, an observed reduction in HIV prevalence and injection risk behavior can be taken as some evidence to support the effectiveness of existing prevention and intervention strategies for HIV/AIDS in the past few years. Our results also underscore the urgent need to develop innovative strategies that can improve accessibility of current harm reduction services, especially to certain subgroups such as migrants, and can incorporate more comprehensive contents including HCV prevention education and other theory-based intervention programs.
